# A Novel Combination Therapy Tβ4/VIP Protects against Hyperglycemia-Induced Changes in Human Corneal Epithelial Cells

**DOI:** 10.3390/bios13110974

**Published:** 2023-11-07

**Authors:** Abdul Shukkur Ebrahim, Thomas W. Carion, Thanzeela Ebrahim, Jeff Win, Hussein Kani, Yuxin Wang, Ashten Stambersky, Ahmed S. Ibrahim, Gabriel Sosne, Elizabeth A. Berger

**Affiliations:** 1 Department of Ophthalmology, Visual & Anatomical Sciences, Wayne State University School of Medicine, Detroit, MI 48201, USA; eabdulsh@med.wayne.edu (A.S.E.); tcarion@med.wayne.edu (T.W.C.); thanze24@wayne.edu (T.E.); phyo.win@med.wayne.edu (J.W.); gp8667@wayne.edu (Y.W.); ashtenstambersky@wayne.edu (A.S.); ahmed.ibrahim@wayne.edu (A.S.I.); gsosne@med.wayne.edu (G.S.); 2 Department of Health Sciences, University of Central Florida College of Health Professions and Sciences, Orlando, FL 32816, USA; husseinkani@gmail.com; 3 Department of Pharmacology, Wayne State University School of Medicine, Detroit, MI 48201, USA; 4 Department of Biochemistry, Faculty of Pharmacy, Mansoura University, Mansoura 35516, Egypt

**Keywords:** human corneal epithelial cells, diabetes, Tβ4, VIP, barrier integrity, tight junction proteins

## Abstract

Despite the prevalence of diabetic retinopathy, the majority of adult diabetic patients develop visually debilitating corneal complications, including impaired wound healing. Unfortunately, there is limited treatment for diabetes-induced corneal damage. The current project investigates a novel, peptide-based combination therapy, thymosin beta-4 and vasoactive intestinal peptide (Tβ4/VIP), against high-glucose-induced damage to the corneal epithelium. Electric cell–substrate impedance sensing (ECIS) was used for real-time monitoring of barrier function and wound healing of human corneal epithelial cells maintained in either normal glucose (5 mM) or high glucose (25 mM) ± Tβ4 (0.1%) and VIP (5 nM). Barrier integrity was assessed by resistance, impedance, and capacitance measurements. For the wound healing assay, cell migration was also monitored. Corneal epithelial tight junction proteins (ZO-1, ZO-2, occludin, and claudin-1) were assessed to confirm our findings. Barrier integrity and wound healing were significantly impaired under high-glucose conditions. However, barrier function and cell migration significantly improved with Tβ4/VIP treatment. These findings were supported by high-glucose-induced downregulation of tight junction proteins that were effectively maintained similar to normal levels when treated with Tβ4/VIP. These results strongly support the premise that Tβ4 and VIP work synergistically to protect corneal epithelial cells against hyperglycemia-induced damage. In addition, this work highlights the potential for significant translational impact regarding the treatment of diabetic patients and associated complications of the cornea.

## 1. Introduction

Despite the prevalence of diabetic retinopathy, up to 70% of the 460+ million diagnosed diabetics worldwide present with signs of diabetic corneal complications [1,2,3,4]. Collectively, these visually disruptive conditions impact the morphological, metabolic, physiological, and clinical aspects of the cornea. Impaired wound healing is the most widely recognized complication, along with diabetic keratopathy. The latter describes several corneal pathologies resulting from chronic hyperglycemia exposure, such as edema, epithelial defects, recurrent erosions, neuropathy with a loss of corneal sensitivity, and tear film changes, which frequently occur in both type 1 and type 2 diabetes mellitus yet often go undiagnosed in most cases [5,6,7]. Notably, dysregulated, delayed corneal wound healing increases susceptibility to corneal ulcers, microbial keratitis, and even corneal perforation [5,8]. However, treatment targeted toward diabetes-induced corneal damage remains limited; thus, further research is warranted to identify potential therapeutic targets.

Barrier integrity of the cornea is largely due to tight junction complexes as they restrict the passage of large molecules and pathogens across the corneal epithelium [9,10,11,12,13]. This complex comprises transmembrane proteins, claudins, occludins, junctional adhesion molecules, and tricellulin; meanwhile, zonula occludens (ZO)-1, ZO-2, and ZO-3 in the peripheral function as scaffolding proteins for intercellular junctions and link transmembrane proteins to the cytoskeleton [11,14]. Compromised epithelial barrier function subsequent to impaired tight junction formation has been indicated as a contributing factor to increased rates of corneal infection in diabetic patients [15,16]. Delayed epithelial wound healing; increased corneal sensitivity; dry eye; corneal edema; and, in some cases, corneal neovascularization can also occur as a result of compromised epithelial barrier function in diabetic patients.

Thymosin-β4 (Tβ4) is a highly conserved 43-amino-acid protein endogenously expressed in nearly all tissues except red blood cells [17,18]. Despite diverse intracellular and extracellular activities, it is widely recognized for its important role in wound healing [19]—a therapeutic aspect that is severely lacking in current treatment options for diabetic corneas. In fact, Tβ4 has been found to promote epithelial cell migration in the cornea, inhibit cell apoptosis by modulating inflammatory cytokine/chemokine release, and influence innate immune cell infiltration and function [20,21,22,23,24,25,26]. Improved wound healing with Tβ4 treatment has been observed in dry eye and alkali burn models [22,27] as well, suggesting that it may confer protection in the diabetic cornea. Moreover, reduced Tβ4 levels have been reported in the cornea of diabetic patients with proliferative diabetic retinopathy [28].

Vasoactive intestinal peptide (VIP) is an endogenous peptide that is produced by neurons and immune cells to function, in part, as an immunomodulator. The potential effects of VIP are not solely due to diabetes-induced enteric nervous system dysfunction, but it has the capacity to function in a more global manner given its widespread distribution [29,30,31,32]. In this regard, previous reports have shown that VIP is protective against high-glucose-induced blood–retinal barrier permeability [33,34], and it was found to decrease VEGF expression in retinal pigment epithelial cells in an in vitro model of diabetic macular edema [35]. In addition, VIP treatment of human retinal endothelial cells significantly reduced high-glucose-induced TNF-α and VEGF levels while increasing the pro-resolving lipid mediator RvD1 and its receptor GPR32 [36]. Activation of pro-resolving circuits such as RvD1 and GPR32 is likely to be beneficial in the context of the diabetic cornea for several reasons, including the resolution of inflammation, and enhanced wound healing and tissue repair.

Both Tβ4 and VIP promote tissue wound healing and regulate inflammation; however, whether they synergistically influence the function of the corneal epithelium under hyperglycemic conditions has yet to be investigated. In the present study, we examined Tβ4 and VIP as a novel, peptide-based combination therapy to prevent high-glucose-induced impairment of corneal epithelial cell barrier function and migration.

## 2. Materials and Methods

### 2.1. Cell Culture of Human Telomerase-Immortalized Corneal Epithelial Cell Line

HUCLs, kindly provided by Dr. Fu-Shin Yu (Wayne State University, Detroit, MI, USA), were previously generated using a retroviral vector that encodes for the human telomerase reverse transcriptase, resulting in immortalization of the cell line [37]. Cells were cultured in DMEM/F-12 (Thermo Scientific, Wyman, MA, USA) supplemented with 10% FBS (Atlantic Biological, Norcross, GA, USA) and 1% penicillin/streptomycin, and maintained in a humidified 5% CO_2_ incubator at 37 °C. HUCLs were used between passages 3 and 5 for all experiments and were authenticated and confirmed to be free from mycoplasma prior to experimentation.

Cell seeding densities were as follows: 60,000 cells/well for ECIS experiments; 300,000 cells/6-well plate for protein analysis; and 300,000 cells/6-well plate containing coverslips coated with fibronectin collagen (FNC Coating Mix, Athena Environmental Service, Inc., Baltimore, MD, USA) for immunostaining. Experimental conditions consisted of normal glucose (5 mM; NG) and high glucose (25 mM; HG) ± Tβ4 (Regenerx Biopharmaceuticals Inc., Rockville, MD, USA), and VIP (Bachem, Torrance, CA, USA). Tβ4 and VIP treatments were administered twice per day to achieve final concentrations of 0.1% and 5 nM, respectively. Mannitol (20 mM) was used as an osmotic control.

### 2.2. Conducting ECIS Experiments for Barrier Function and Cell Migration

Barrier function and cell migration were determined using the ECIS Zϴ system (Applied Biophysics Inc., Troy, NY, USA). Each array (Applied Biophysics Inc.) consists of a 96-well plate, with each well containing either an electrode with an inter-digitated finger configuration (total electrode area = 3.92 mm^2^) for barrier function (array 96W20idf) or two circular 350 µm diameter electrodes (total electrode area = 0.256 mm^2^) for cell migration (array 96W1E+). The arrays were pre-treated as previously described [38]. Before inoculation, electrode impedance values were stabilized to reduce electrode drift. After seeding, the HUCLs were switched to serum-free media 12–16 h prior to initiating experimental conditions.

Plates were maintained at a constant current of ~1 µA. The runs were carried out under multiple frequencies (1000 Hz–64 kHz) and monitored continuously with measurements obtained ~2 min apart. Barrier function assessments were carried out for 120 h. For cell migration, an automated wound was induced (wound time—60 s, current—2800 µA, frequency—60 kHZ), producing two cell-free areas within the cell monolayer. Cell migration assessments were carried out for a duration of 70 h.

### 2.3. Data Analysis and Modeling

ECIS measurements were obtained as previously reported [38] for overall resistance (4 kHz), impedance (32 kHz), and capacitance (64 kHz) as a function of time. Cell data were normalized by comparison with cell-free electrodes. In addition, impedance (*y*-axis) was measured as a function of frequency (*x*-axis) and time (*z*-axis), and represented as a 3D plot.

Mathematical modeling was applied to derive three parameters: R_b_ (resistance between cells, Ω-cm^2^), α (basolateral resistance between cells and underlying substrate, Ω-cm^1/2^), and c_m_ (cell membrane capacitance, µF/cm^2^). To validate the modeled data, Drift Correction and Model Fit RMSE (root mean square error) values were used. The migration velocity in response to wounding was calculated by dividing the total distance that the cells migrated over the electrode radius (175 µm) by the time required to fully recover the normalized resistance prior to wounding.

### 2.4. Western Blot Analysis

After six days of experimental conditions, cellular lysates were prepared using RIPA buffer (Cell Signaling Technology, Danvers, MA, USA) containing phosphatase and protease inhibitors (Thermo Fisher Scientific, Waltham, MA, USA), then centrifuged at 14,000× *g* for 15 min. Supernatants were further normalized to ensure equal amounts of protein using the BCA method (Thermo Scientific). Subsequently, samples were separated onto 4–20% tris-glycine gels (Invitrogen, Carlsbad, CA, USA) and transferred to PVDF membranes. Next, membranes were blocked using 5% non-fat milk dissolved in TBST (10 mM Tris-HCl buffer, pH 8.0, 150 mM NaCl, and 0.1% Tween 20) at room temperature for 30 min. Membranes were then incubated at 4 °C overnight with antigen-specific primary rabbit antibodies as follows: anti-ZO-1 (1:500; Cat #40-2200, Invitrogen, Waltham, MA, USA), anti-ZO-2 (1:500; Cat #2847), anti-occludin (1:500; E6B4R, Cat #91131), anti-claudin-1 (1:500; Cat #4933) (Cell Signaling Technology, Danvers, MA, USA), and anti-β-actin (1:1000; C4; Cat #sc-47778; Santa Cruz Biotechnology, Dallas, TX, USA). Each blot was incubated with goat anti-rabbit HRP-conjugated secondary antibodies (Cat #111-035-144; Thermo Fisher Scientific) for 1 h at room temperature, followed by ECL Chemiluminescent Substrate incubation (Thermo Fisher Scientific). After detection of ZO-1, ZO-2, occludin, and claudin-1, Restore PLUS Western blot stripping buffer (Thermo Fisher) was used to strip PVDF membranes (15 min at room temperature), which were then re-probed for β-actin (internal control). Western blot images were collected (Bio-Rad Molecular Imager, ChemiDoc XRS+; Azure Imaging System AI600-1360) and analyzed using Image Studio Lite software version 5.2 (LI-COR Biosciences, Lincoln, NE, USA). Target protein expression was quantified after normalizing to β-actin.

### 2.5. Immunostaining of Tight Junction Molecules

Barrier integrity of the epithelium was qualitatively evaluated through ZO-1 and occludin immunostaining. Changes in cellular proliferation were determined by Ki-67 immunostaining. After five days, cells were confirmed to be confluent before coverslips were fixed (Z-Fix; Anatech Ltd., Battle Creek, MI, USA) for 15 min at room temperature. Next, cells were permeabilized (0.05% triton X-100, 15 min on ice) and rinsed (PBS, 2×), followed by blocking (5% BSA, 1 h). Cells underwent overnight incubation at 4 °C with anti-ZO-1 rabbit mAb Alexa Fluor 488 Conjugate (1:100) (D6L1E; Cat #86942; Cell Signaling Technologies), FITC-conjugated anti-occludin mouse mAb (1:200) (OC-3F10; Cat #33-1511; Invitrogen, Eugene, OR, USA), or FITC-conjugated anti-Ki-67 mouse mAb (1:100) (Cat #556026; BD Biosciences, San Diego, CA, USA). Each coverslip was rinsed and mounted using Prolong Diamond plus DAPI (Invitrogen, Waltham, MA, USA). Final images were taken with an Olympus DP72 microscope with a 40× objective lens UApo N 340 40×/1.35 Oil. Using cellSens dimensions software (cellSens Dimension_1_18), images were captured with a 360 nm filter set to view DAPI (blue) and 488 nm filter set to view FITC (green). Images for green and blue channels in each group were overlapped using ImageJ 1.44 software (NIH, Bethesda, MD, USA).

### 2.6. Statistical Analysis

ECIS assays were conducted in five independent experiments, and Western blot and ICC assessments from at least three independent experiments. ECIS data are presented as mean ± SEM, and Western blot data are presented as mean ± SD. All data were analyzed by a one-way ANOVA followed by Bonferroni’s multiple comparison test (GraphPad Prism; San Diego, CA, USA). Data were considered statistically significant at *p* ≤ 0.05. Unless indicated differently, the data shown are representative of a typical experiment. Group sizes, determined prior to experimentation, are indicated in the figure legends.

## 3. Results

### 3.1. Bioimpedance Analysis of Barrier Function

**Figure 1:**
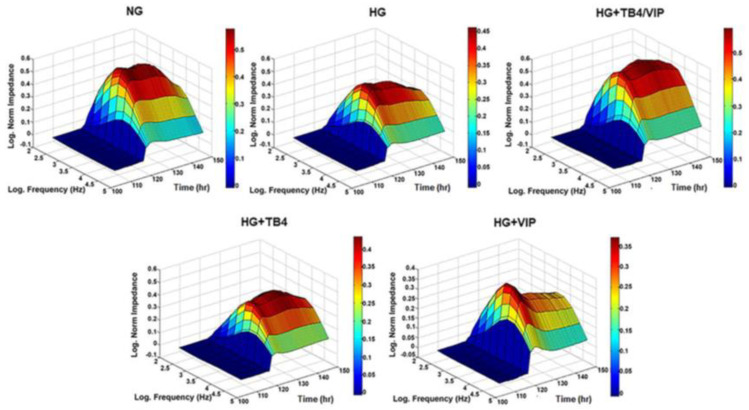
Barrier function of HUCLs reflected by real-time bioimpedance analysis using an AC frequency scan. HUCLs were seeded (60,000 cells/well) on a 96W20idf ECIS array. Three-dimensional representation of the log-normalized impedance (*y*-axis) as a function of the log frequency of both AC (*x*-axis) and time (*z*-axis). Cells maintained in NG, HG, HG + Tβ4/VIP (**top row**), HG + Tβ4, and HG + VIP (**bottom row**) are represented. Time = 0 denotes time of inoculation. Log-normalized impedance values are also represented by the color bar.

The influence of hyperglycemic conditions and peptide treatment was assessed by bioimpedance analysis, as shown in Figure 1. The formation of a mature confluent barrier is demonstrated by the plateau in impedance, shown as log-normalized values on the *y*-axis. Cells maintained in NG reached a higher impedance value (~0.4 Ω) compared with HG (~0.3 Ω). Of the different treatment groups exposed to HG, only the combination therapy Tβ4/VIP (~0.45 Ω) maintained impedance values similar to NG. On the other hand, Tβ4 and VIP monotherapies (~0.3 and 0.25 Ω, respectively) remained comparable to HG conditions.

### 3.2. Barrier Function Measurements

**Figure 2:**
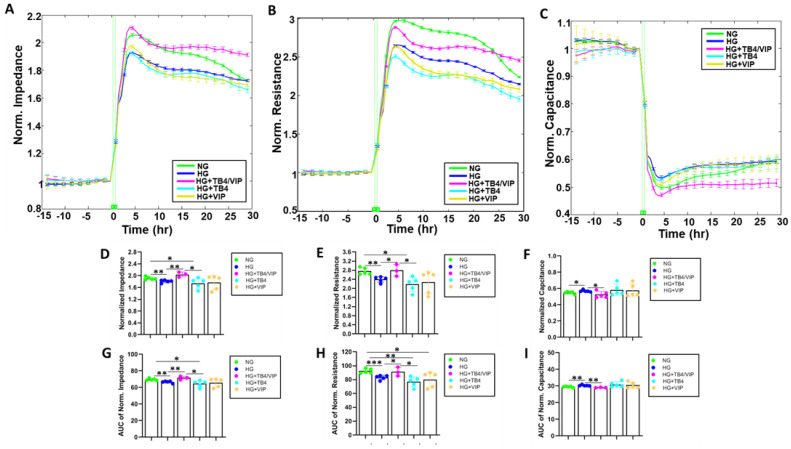
Real-time monitoring of HUCL impedance (measured at an AC frequency of 32 kHz) (**A**), resistance (measured at an AC frequency of 4000 Hz) (**B**), and capacitance (measured at an AC frequency of 64 kHz) (**C**) on a 96W20idf ECIS array. Cells at a 60,000 seeding density were maintained in NG, HG, HG + Tβ4/VIP, HG + Tβ4, and HG + VIP. Impedance, resistance, and capacitance were measured from the time of inoculation (T = 0 h) to 120 h after treatment application. Bar graph representation of normalized impedance (**D**,**G**), resistance (**E**,**H**), and capacitance (**F**,**I**) endpoint values and area under the curve, respectively, for each group. Data shown are mean ± SEM; n = 5/group. * *p* ≤ 0.05; ** *p* ≤ 0.01; *** *p* ≤ 0.001.

Impedance, resistance, and capacitance plot tracings are shown in Figure 2A–C, respectively. HUCLs maintained in HG displayed significantly lower Impedance (D,G), resistance (E,H) and capacitance (F,I) are shown as endpoint and area under the curve (AUC) values. HUCLs maintained in HG displayed significantly lower impedance and resistance and significantly higher capacitance when compared with cells in NG, indicating that the corneal epithelial barrier under NG conditions is not as tight or strong comparatively.

In addition, only Tβ4/VIP-treated HUCLs prevented the HG-induced decreases in both impedance (D,G) and resistance (E,H). No effect, however, was observed regarding either parameter after Tβ4 or VIP monotherapy when exposed to HG. Similarly, only Tβ4/VIP-treated HUCLs mitigated HG-induced increases in capacitance (F,I) values, while no differences were observed with either monotherapy. Taken together, only the combination Tβ4/VIP therapy was able to maintain the characteristics of these cells despite HG conditions.

### 3.3. Mathematical Modeling Parameters of the R Data—α, R_b_, and C_m_

**Figure 3:**
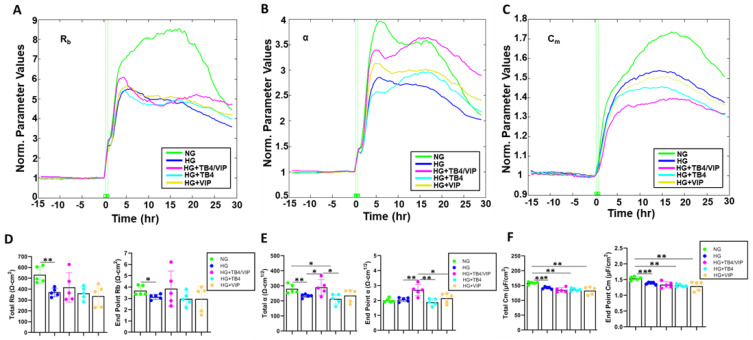
Mathematical modeling of R_b_, α, and C_m_ for HUCLs maintained in NG, HG, HG + Tβ4/VIP, HG + Tβ4, and HG + VIP. Modeled normalized parameters R_b_ (**A**), α (**B**), and C_m_ (**C**) were traced over 120 hr. Bar graphs represent total and endpoint values as calculated for R_b_ (**D**), α (**E**), and C_m_ (**F**). Data shown are the mean ± SD; n = 5/group. * *p* ≤ 0.05; ** *p* ≤ 0.01; *** *p* ≤ 0.001.

One of the benefits of the ECIS software is the ability to model the impedance (Z) into parameters that distinguish between cell–cell (R_b_) and cell–matrix (α) adhesions, as well as membrane capacitance (C_m_). R_b_, α, and C_m_ were extrapolated from the generated impedance data and are presented as plot tracings in Figure 3A–C, respectively. Both total and endpoint values for R_b_ (D), indicative of paracellular barrier strength, were significantly decreased in HUCLs maintained in HG compared with NG conditions. Total α, indicating the strength of interaction between the HUCLs and the basal substrate, was also significantly lower with HG exposure (E), despite no difference in endpoint values. Further, both total and endpoint C_m_ values (F), used to determine if changes in the capacitance are a result of changes in electrode coverage or are a function of microvariations in the apical membrane structures, were also significantly decreased in cells maintained in HG compared with NG.

Only the combination Tβ4/VIP treatment resulted in a significant increase in α values (both total and endpoint) compared with HG (E), whereas no differences were detected for R_b_ or C_m_ (panels D and F, respectively). No differences were observed for either monotherapy compared with HG. Thus, these data indicate that Tβ4/VIP treatment results in stronger cell–matrix interactions despite HG conditions.

### 3.4. Bioimpedance Analysis of Wound Healing Response

**Figure 4:**
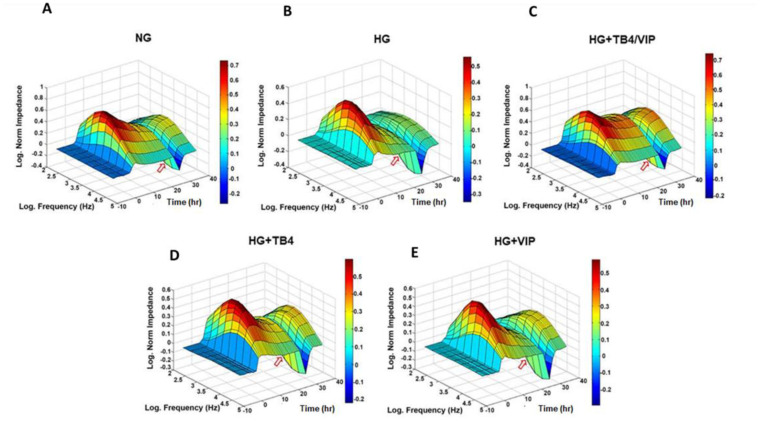
Cell migration/electroporation wounding of HUCLs monitored by real-time bioimpedance analysis using an AC frequency scan. HUCLs were seeded (60,000 cells/well) on a 96W1+ ECIS array. 3D representation of the log-normalized impedance (*x*-axis) as a function of the log frequency of both AC (*y*-axis) and time (*z*-axis). Cells maintained in NG (**A**), HG (**B**), HG + Tβ4/VIP (**C**), HG + Tβ4 (**D**), and HG + VIP (**E**) are represented. Wound induction is denoted by the red arrow. Dark red peaks reflect a media change. Time = 0 denotes time of inoculation. Log-normalized impedance values are also represented by the color bar.

The wound healing response was next assessed by ECIS to further investigate the efficacy of Tβ4/VIP treatment. Figure 4A–E shows the 3D bioimpedance analysis of cell migration and wounding for NG and HG ± Tβ4 and VIP. Once a functional barrier was in place (plateau), a high field pulse was induced (red arrow), producing a wound, represented by the drop (valley) in normalized impedance. HUCLs maintained in NG reestablished a stronger barrier after wounding compared with those in HG, as evidenced by the log-normalized impedance values of ~0.3 Ω for NG (A) and ~0.1 Ω for HG (B). HUCLs maintained in HG + Tβ4/VIP (C) recovered to ~0.4 Ω, greater than that of NG and both Tβ4 (~0.3 Ω) (D) and VIP (~0.25 Ω) (E) monotherapies.

### 3.5. Wound Healing Measurements

**Figure 5:**
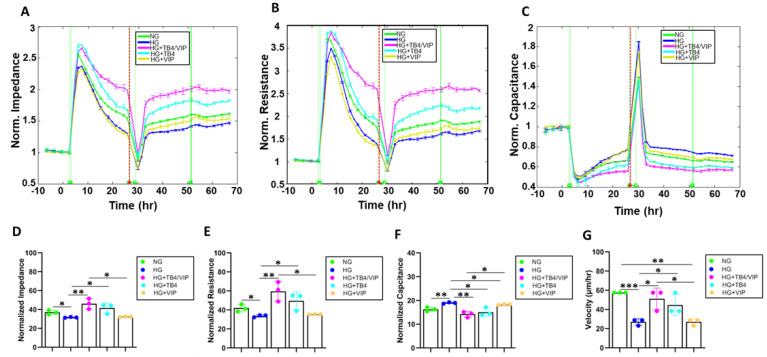
Real-time monitoring of HUCL impedance (measured at 32 kHz) (**A**), resistance (measured at 4000 Hz) (**B**), and capacitance (measured at 64 kHz) (**C**) on a 96W1+ ECIS array. Cells at a 60,000 seeding density were maintained in NG, HG, HG + Tβ4/VIP, HG + Tβ4, and HG + VIP. Impedance, resistance, and capacitance were measured from the time of wounding (T = 30 h) to 70 h post-wound. Bar graphs represent total values as calculated for normalized impedance (**D**), resistance (**E**), capacitance (**F**), and cell velocity (µm/h) (**G**), presented for each group. Data shown are the mean ± SEM; n = 5/group. * *p* ≤ 0.05; ** *p* ≤ 0.01; *** *p* ≤ 0.001.

To further assess the wound healing response of HUCLs maintained in HG and the therapeutic effects of the combination Tβ4/VIP treatment, normalized impedance, resistance, capacitance, and wound velocity were determined (Figure 5A–G). HG conditions significantly reduced both impedance (A,D) and resistance (B,E) after wounding compared with NG conditions, while capacitance (C,F) was significantly elevated. In addition, the cell velocity (G) was significantly reduced following wounding of HUCLs maintained in HG compared with those in NG.

Similar to the effect observed with barrier function, the combination of Tβ4/VIP significantly increased impedance, resistance, and cell velocity; capacitance was significantly decreased despite the HG conditions. Given the powerful wound healing properties associated with Tβ4, it was not surprising that the Tβ4 monotherapy also significantly elevated impedance, resistance, and cell velocity, along with significantly decreasing capacitance. No differences were observed between impedance, resistance, capacitance, or cell velocity for the VIP monotherapy compared with HG; however, capacitance was significantly reduced.

### 3.6. Western Blot Analysis of the Tight Junction Protein Complexes

**Figure 6:**
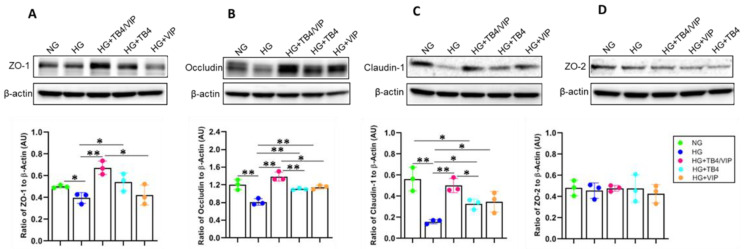
Detection of select tight junction complex components as detected by Western blot in HUCLs maintained in NG, HG, HG + Tβ4/VIP, HG + Tβ4, and HG + VIP. Protein levels for ZO-1 (**A**), occludin (**B**), claudlin-1 (**C**), and ZO-2 (**D**) are presented as a ratio to β-actin ± SD. n = 5/group. * *p* ≤ 0.05; ** *p* ≤ 0.01.

As an extension of the ECIS study, alterations in tight junction proteins ZO-1, ZO-2, claudin-1, and occludin were also determined. As shown in Figure 6, protein levels for ZO-1 (A), occludin (B), and claudin-1 (C) were significantly reduced in HUCLs maintained under HG compared with those under NG conditions. Remarkably, Tβ4/VIP significantly upregulated all three molecules compared with HG-treated cells. Somewhat unexpectedly, the Tβ4 monotherapy also resulted in a significant increase in ZO-1, occludin, and claudin-1, while the VIP monotherapy significantly upregulated levels of occludin and claudin-1 compared with HG. No effects were observed between any of the groups regarding ZO-2. Taken together, these results reveal that Tβ4/VIP treatment is a more efficacious therapy than either single-peptide monotherapy, which strongly suggests that Tβ4 works synergistically with VIP to enhance the observed functional effect.

### 3.7. IHC Assessment of the Tight Junction Protein Complexes

**Figure 7:**
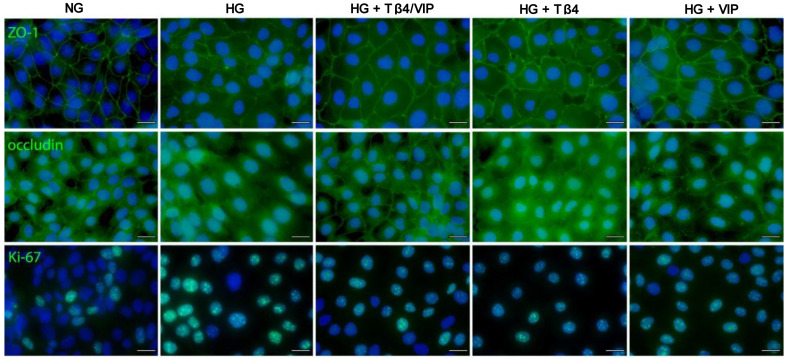
Tight junction staining in HUCLs with DAPI nuclear stain. Positive immunostaining of ZO-1 (**top row**) and occludin (**middle row**) is shown in green, highlighting the localization of tight junctions. DAPI staining (blue) provides visualization of the cellular nuclei. Ki67 staining (green) (**bottom row**) indicates the presence of the Ki-67 protein within the nucleus of cells undergoing active proliferation. Magnification = 40×. Scale bar = 20 µm.

IHC was carried out to detect ZO-1 and occludin expression in HUCLs maintained in either NG or HG ± Tβ4/VIP treatments (Figure 7). Under NG conditions, ZO-1 staining (top row) presented specifically along the border of each cell, highlighting the cobblestone-like appearance of epithelial cells. There were no observable gaps or indications of membrane swelling. Occludin staining (middle row) displayed a similar pattern under NG conditions. There was positive staining along the plasma membrane, reflecting that occludin is localized to the intercellular junctional complexes. While it is mainly localized at tight junctions, some positive staining was observed intracellularly. Both ZO-1 and occludin staining intensities were lower in HUCLs maintained in HG compared with those in NG. The continuous linear pattern of ZO-1 and occludin staining associated with intercellular junctional complexes appears to be markedly reduced with HG exposure. Single channel images are available as Supplementary Material (Figure S1). 

Additionally, HUCLs maintained in HG appeared to be more flattened or elongated compared with the normal cobblestone-like morphology, with fewer cell–cell interactions, as reflected by ZO-1 and occludin staining. Although Tβ4 and VIP monotherapies improved the intensity of both ZO-1 and occludin staining, the cell morphology remained somewhat flattened and elongated, whereas combination Tβ4/VIP treatment of HUCLs maintained under HG conditions resulted in stronger staining of ZO-1 as a distinct band-like pattern at the cell borders with more punctate staining outlining the boundaries between adjacent cells. Occludin staining was more intense, not only reflecting a similar band-like pattern along cell borders, but also more positive staining intracellularly, likely reflecting the trafficking dynamics and cellular processes influenced by HG conditions.

### 3.8. HUCL Cell Proliferation

HUCLs were also stained for the proliferation marker Ki-67 (Figure 7, bottom row) to determine whether the observed changes were due, in part, to differences in cellular proliferation rates. Positive Ki-67 staining was observed as nuclear with a punctate appearance in all groups. However, HUCLs maintained in HG appeared to have more Ki67^+^ cells and more intense staining when compared with those in NG, while the Tβ4/VIP, Tβ4, and VIP treatment groups demonstrated staining patterns comparable to NG despite HG exposure, indicating similar proliferation rates between the three treatment groups and the NG controls.

## 4. Discussion

Diabetic corneal complications, including impaired epithelial barrier function, delayed wound healing, and increased susceptibility to infection, pose significant challenges in the management of diabetic patients. These complications can lead to vision impairment, severely impacting the quality of life for individuals with diabetes. Despite advances in diabetes management, there remains a critical need for effective treatments targeting corneal complications. In this study, we investigated the potential therapeutic efficacy of Tβ4/VIP in mitigating diabetic corneal complications by evaluating its effects on corneal epithelial barrier function and wound healing.

Our previous work illustrated that Tβ4 influences multiple wound healing pathways in the cornea, including fibronectin:integrin and uPA:uPAR [23]. In the clinical setting, Tβ4 treatment has been shown to reduce non-healing epithelial defects in diabetic patients with neurotrophic keratopathy [39]. Our work in a bacterial keratitis model also revealed that VIP treatment modulates cytokine/chemokine production, decreases inflammatory cell infiltration, and enhances growth factor production and β-defensins levels, subsequently promoting healing and tissue restoration in the infected cornea [32,40,41]. The Yu lab linked defects in corneal epithelial cell–dendritic cell–sensory nerve interactions to the pathogenesis of neurotrophic keratopathy [42,43,44], while also showing that VIP partially restored corneal epithelial wounds after debridement in an STZ-induced T1DM mouse model [45]. Although Tβ4 and VIP have been investigated as monotherapies in the eye, it is unknown whether the two molecules can work synergistically to further improve the pathogenic hyperglycemia-induced effects on corneal epithelial cells.

Use of ECIS biosensor technology allows insight into key aspects of corneal epithelial cellular behavior in response to HG exposure and how this response is altered in the presence of Tβ4/VIP combination treatment while also replacing the use of animals in research. Impedance is the overall opposition to the AC flow. Monitoring changes in impedance over time provides insights into various cellular processes, such as cell adhesion, spreading, proliferation, migration, and barrier integrity. Impedance measurements by ECIS reflect alterations in cell morphology, attachment, and interactions with the substrate. The observed decrease in impedance in cells maintained in HG indicates cell detachment or loss of cell–substrate contacts, whereas increased impedance with Tβ4/VIP treatment signifies that cell spreading, proliferation, or the formation of cell–cell contacts have been maintained despite HG exposure.

Impedance is a value that consists of both resistance and capacitance. Resistance represents the opposition to the flow of electrical current and is a measure of the integrity and tightness of the corneal epithelial barrier. It reflects resistance to the passage of ions and molecules through the paracellular pathway between adjacent cells. The higher resistance values observed under NG conditions indicate a more intact and tightly sealed barrier, with reduced paracellular permeability. The reduced resistance values obtained from HG conditions suggest reduced cell–substrate adhesion, loss of barrier integrity with increased paracellular permeability, cell detachment or loss of viability, and altered cellular morphology. These data coincide with previous findings that HG conditions reduce the barrier integrity of corneal epithelial cells [15], retinal pigment epithelial cells [45], and intestinal epithelial cells [45,46]. Remarkably, Tβ4/VIP prevented these HG-induced changes in the barrier integrity and function of human corneal epithelial cells.

Lower membrane capacitance values, as observed in HUCLs maintained under NG conditions, reflect tighter and more stable cell–cell contacts at tight junctions. They also indicate increased electrical coupling between adjacent corneal epithelial cells. Although compromised when cells were maintained under HG conditions, Tβ4/VIP effectively prevented these pathogenic changes. As a result, cell–cell communication is maintained, facilitating coordinated cellular responses, such as the synchronization of cell behavior and signaling within the epithelium, which can be important for maintaining tissue function and integrity.

Mathematical modeling revealed that HG lowered R_b_—indicating alterations in cell–substrate interactions and changes in cell morphology; lowered α—suggesting changes in cell adhesion, spreading, and migration or changes in cell–substrate interactions; and increased C_m_—a reflection of morphological changes, alterations in membrane dynamics, or changes in the protrusions or extensions of the cell surface. Tβ4/VIP effectively strengthened adhesion between the cells and the substrate, indicating better cell attachment and spreading; however, cell–substrate interactions and changes in cellular morphology likely contributed to the overall differences observed in the resistance data.

Regarding the wound healing process, as the corneal epithelial cells migrate and cover the cell-free region, impedance gradually increases, reflecting the restoration of cell–substrate adhesion, reformation of tight junctions, and reestablishment of the epithelial barrier. This is observed in cells maintained in NG, and was markedly impaired in HUCLs exposed to HG, as indicated by the impedance, resistance, capacitance, and cell velocity changes. These results correlate with a previous in vivo study showing that cells in high glucose have weaker electric fields and migrate slower [47]. Remarkably though, the impaired response under HG conditions was fully mitigated in cells exposed to HG + Tβ4/VIP. These data demonstrate that Tβ4/VIP effectively facilitates the migration and proliferation of corneal epithelial cells during the healing process, contributing to efficient closure of the wound and restoration of corneal integrity even under HG conditions.

The assessment of tight junction complexes is integral to understanding the function and dynamics of corneal epithelial cells. The assembly of tight junction protein molecules, such as occludin, claudins, and ZO-1, plays a crucial role in maintaining the integrity and selective permeability of the corneal epithelial barrier [10,11,12]. ZO-1 is a cytoplasmic protein that acts as a scaffolding protein, linking transmembrane tight junction proteins, such as occludin and claudins, to the actin cytoskeleton. Our selection of claudin-1 was based on previous reports demonstrating that it was expressed in corneal and conjunctival epithelial cells, while claudin-2 and claudin-3 were undetectable [9,48]. The PDZ domain in claudins exerts its anchoring function by interacting with the intracellular ZO molecules and connecting to cytoskeletal proteins [49,50]. Occludin is an integral membrane protein that contributes to tight junction stabilization, cell signaling and regulation, cell polarity, and optimal barrier function [51]. It interacts with claudins and ZO-1 to form the sealing strands of the tight junction complex. Our findings demonstrating that tight junction proteins were significantly reduced under HG conditions align with prior work in the field [15,52]. Interestingly, all three treatments—combination Tβ4/VIP, and Tβ4 and VIP monotherapies—effectively prevented HG-induced decreases in ZO-1, occludin, and claudin-1 with significant increases noted with Tβ4/VIP. A previous study showed that Tβ4 significantly improved vascular permeability dysfunction and regulated tight junction protein stability [53]. Scuderi et al. reported that treatment with VIP significantly restored both claudin-1 and ZO-1 expression, and increased barrier integrity, in hyperglycemia-induced ARPE-19 cells [34]. Our findings support these previous results and provide further evidence for a synergistic effect between Tβ4 and VIP in restoring tight junctions, which is thought to be a driving factor for the observed functional differences detected by ECIS.

Immunostaining confirmed the breakdown of tight junction complexes under HG conditions. All three treatment groups (Tβ4/VIP, Tβ4, VIP) maintained the expected continuous, linear pattern along cell–cell contacts, with the most intense positive staining observed in cells treated with Tβ4/VIP. While occludin is mainly localized at the tight junctions, a small fraction of occludin has been observed in intracellular compartments (endosomes, Golgi apparatus), where it is thought to regulate the assembly, trafficking, and turnover of tight junctions. The more intense intracellular occludin^+^ staining could also be due to endocytosis. Occludin can be internalized through clathrin-mediated or caveolae-mediated endocytosis, then transported to endosomes or lysosomes, where it can undergo degradation or recycling. Another possibility is that it may be temporarily internalized and sequestered intracellularly to allow for dynamic changes in cell shape and movement. Regardless, it is worth further investigation. It was also noticed that HG conditions altered epithelial cell shape and morphology, presenting with a more flattened or elongated appearance compared with the classic cobblestone-like morphology observed under NG conditions. This correlates with the C_m_ values that indicate changes in membrane structure in response to HG exposure. This morphological change was also observed in HUCLs exposed to HG but treated with Tβ4 or VIP as monotherapies, albeit less severe. Cell morphology was notably maintained in the Tβ4/VIP-treated cells despite HG exposure. Further, only with the combination treatment did the cell morphology and protein expression of ZO-1 and occludin most resemble those observed under NG conditions.

Our complementary approach provides a foundation for the therapeutic effects of Tβ4/VIP regarding hyperglycemia-induced changes in corneal epithelial cell behavior, barrier integrity, and wound healing mechanisms. HG conditions have been associated with alterations in tight junction integrity and function, compromising the corneal epithelial barrier. When administered as a combination therapy, these bioactive peptides effectively mitigate the HG-induced disruptions in tight junction complexes, preserving the integrity and functionality of the corneal epithelial barrier, even during the wound healing response. However, it is prudent to acknowledge that this work was carried out using an in vitro cell culture model, which does not fully capture the complexity of in vivo conditions. As an extension of this point, the current study focuses on human corneal epithelial cells that play a major role in the structure and function of the cornea, but do not represent the entire cellular diversity and responses within the diabetic cornea. In addition, while simplified high-glucose conditions mimic hyperglycemia, the diabetic cornea is affected by multiple factors, including inflammation and oxidative stress, which are not fully replicated in the current model. That being said, future directions will include in vivo studies to validate the findings observed in vitro. Diabetic animal models will not only provide a more comprehensive understanding of the effects of Tβ4/VIP on the diabetic cornea in a physiological context, but also allow for the mechanistic exploration of our combination peptide treatment. Longitudinal studies will also provide insights into the sustained effects of Tβ4/VIP. Overall, the combined therapeutic potential of Tβ4/VIP offers a promising strategy to counteract the detrimental effects of HG on the corneal epithelium and warrants further investigation into its protective role regarding the diabetic cornea and related complications.

## Data Availability

Data sharing is not applicable to this article as no datasets were generated or analyzed during the current study.

## Supplementary Materials

The following supporting information can be downloaded at: https://www.mdpi.com/article/10.3390/bios13110974/s1, Figure S1: Merged images from Figure 7 are provided herein as single channel images, illustrating the localization of tight junction proteins, ZO-1 (top) and occludin (middle), along with Ki-67 (bottom) as a marker of cellular proliferation. DAPI nuclear stain is shown in blue. Magnification = 40×.

## Abbreviations

ECIS	electric cell–substrate impedance sensing
HG	high glucose (25 mM)
HUCLs	human telomerase-immortalized corneal epithelial cells
NG	normal glucose (5 mM)
ZO	zonula occludens
