# Supplementary material for: A Novel Combination Therapy Tβ4/VIP Protects against Hyperglycemia-Induced Changes in Human Corneal Epithelial Cells
Source: Biosensors (Basel). 2023 Nov 7;13(11):974. doi: 10.3390/bios13110974 (PMC10669755; doi:10.3390/bios13110974)
Supplement: Supplementary file 1 [file biosensors-13-00974-s001.zip › biosensors-2585345-supplementary.pdf]

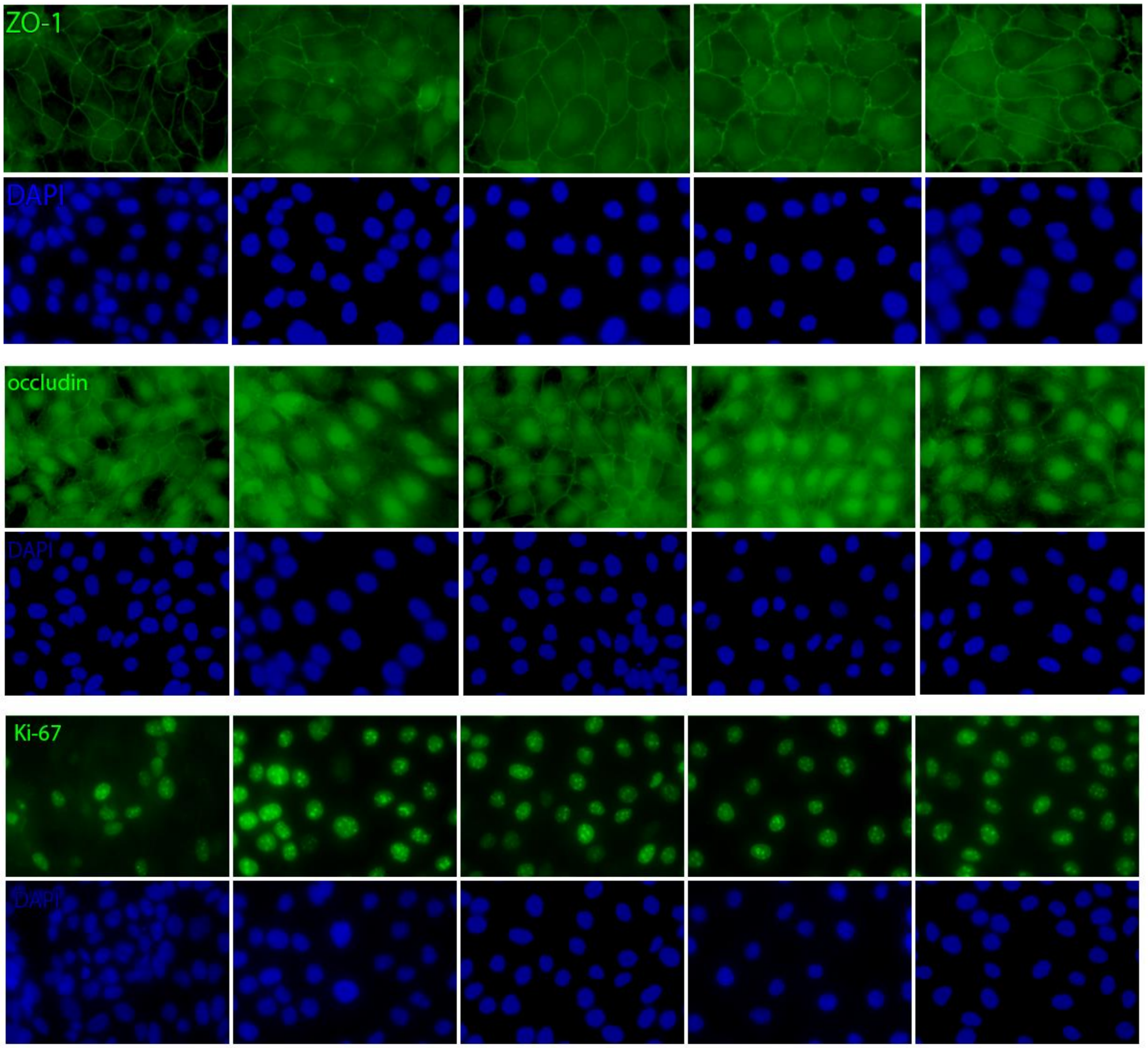

Supplementary Figure S1. Merged images from Figure 7 are provided herein as single channel images, illustrating the localization of tight junction proteins, ZO-1 (top) and occludin (middle), along with Ki-67 (bottom) as a marker of cellular proliferation. DAPI nuclear stain is shown in blue. Magnification = 40x.
